# Supplementary material for: A micro-PRNT for the detection of Ross River virus antibodies in mosquito blood meals: A useful tool for inferring transmission pathways
Source: PLoS One. 2020 Jul 24;15(7):e0229314. doi: 10.1371/journal.pone.0229314 (PMC7380888; doi:10.1371/journal.pone.0229314)
Supplement: Attachment — Submitted filename: Response to Reviewers_Gyawali et al_ 050620.docx [file pone.0229314.s001.docx]

**Manuscript ID PONE-D-20-02979 Response to Reviewers**

**Title**: A micro-PRNT for the detection of Ross River virus antibodies in mosquito blood meals: a useful tool for inferring transmission pathways.

**Comments to the Editor**

We have amended the ethics statements according to your instructions. Written consent was required by volunteers. That information is now included in the last paragraph of the section “Preparation of blood fed mosquitoes”.

We have made a number of changes to the text, additional to the requests may by the reviewers. These are visible in the track changed R1 manuscript. There have been no changes in context, methodology or interpretation but we have altered sections of text for clarity or to correct grammar. The major changes are detailed below:

1. Introduction: We have moved two paragraphs form the end of the introduction to further up the page.

2. Preparation of blood fed mosquitoes: First and last paragraphs revised for methodological clarity and to include requested information on ethics.

3. Validation of the micro PRNT: We have clarified some of information on blood meal volumes.

4. The text in the discussion has been improved for flow and clarity, but the interpretation of results has not changed.

**Response to Reviewers**

**Reviewer #1**

**Comment 1**: Interesting technical paper with only minor edits.

Line 70 use of word "encourage" needs to be re-examined

Line 91 West Nile viruses

Lines 233 and 241 missing period.

Line 302 sometimes

**Response**: We thank the reviewer for their positive assessment of the study and for identifying these minor edits. These have been corrected in the updated manuscript.

**Comment 2**: Overall - I am not sure how useful some of the figures are. Figure 1 needs T=0 or state it was fully engorged - even with this I am not sure it really adds anything.

Figure 4 B and C - so small hard to see better to summarize data and leave the plates out?

**Response**: We feel that Fig 1 is a useful visual representation of the amount of blood present at various times post feeding. We have updated Fig 1 to include 0 (fully engorged) and 6h post feeding.

For Fig 4, we agree with the reviewer. We have removed the photos of the micro-titre plates, leaving only the line graph to demonstrate the relationship between antibody concentration and plaque inhibition over time.

**Reviewer #2**

**Comment 1:** The work proposes the use of micro PRNT from blood present in mosquito abdomens, useful tool for viral ecology projects, standardized in that study for Ross River virus. Very important and well-designed work.

Line 96 – What is "ca"? Appears on line 305 again.

Line 107 – The reagent "L-glutamine (200 mM)" is repeated in the formulation. I believe it must be an antimicrobial, perhaps Gentamicin.

Lines 171 – 172 “The volume of RPMI 1640 was adjusted to obtain a 20-fold dilution of the blood.” What level of volume control is there in this process? Is the blood volume measured? That part was not clear.

Lines 180 – 181 “Aedes notoscriptus that had been fed on human seropositive or seronegative volunteers” Mention the quality control that is done on these mosquitoes, as they were used to feed on human volunteers.

The legends of figures 1, 2 and 3 are incomplete. In all of them the information needs to be complemented.

**Response:** We thank the reviewer for their positive assessment of the study.

“ca” (Circa) has been replaced with “approximately”.

We have removed reference to L-glutamine which is an amino acid and a common additive to cell culture media.

We used fully engorged mosquitoes, published estimates of mean blood volumes (e.g Konishi 1989, Jeffery 1956) and applied dilution factors to those published estimates.

All mosquitoes were from established, uninfected colonies. The sero-status of human volunteers was confirmed by standard PRNT. Each blood-feed lasted until engorgement or termination (30 minutes). Only mosquitoes that were fully engorged were selected for further analysis. Three engorged mosquitoes per time point were chosen for analysis.

We have attempted to make this process clearer in the methods section under ‘Preparation of blood fed mosquitoes’.

The legends for figures 1, 2 and 3 have been expanded.

**Comment 2:** There is a lack of discussion topic of the complexity of using the tool in fieldwork regarding the possible species involved as hosts. According to the protocol described here, the volume would be enough to screen how many species? Does the protocol allow a focused or broad-spectrum host approach? The proof of concept was made with blood obtained from human and sheep, but how would be the use of the protocol in other works, considering that this is the final objective of the publication.

**Response**: We believe that we have already acknowledged the complexity in interpretation in our discussion, having referred to sampling bias, cross-reactivity, inability to identify all potential vertebrate hosts and the fact that the presence of antibody in a mosquito blood meal does not necessarily define that mosquito as a key vector.

Our proof of principle shows that the same mosquito blood meals can be used to identify the host origin of the blood meal and whether that host had been exposed to a target virus (in this case the arbovirus RRV). The technique provides clues to potential transmission pathways, but is not definitive.

We agree with the reviewer that a broad spectrum of hosts might be represented in mosquito blood meals. Although we used human and sheep blood for demonstration purposes, sequencing data from a single, tiny aliquot of serum can be matched to myriad vertebrate sequences available online.
